# Specific effects of antitumor active norspermidine on the structure and function of DNA

**DOI:** 10.1038/s41598-019-50943-1

**Published:** 2019-10-18

**Authors:** Takashi Nishio, Yuko Yoshikawa, Chwen-Yang Shew, Naoki Umezawa, Tsunehiko Higuchi, Kenichi Yoshikawa

**Affiliations:** 1 Faculty of Life and Medical Sciences, Doshisha University, Kyotanabe, 610-0394 Japan; 2 Doctoral Program in Chemistry, The Graduate Center of the City University of New York, New York, 10016 USA; 3 Department of Chemistry, College of Staten Island, Staten Island, New York, 10314 USA; 4 Graduate School of Pharmaceutical Sciences, Nagoya City University, Nagoya, 467-8603 Japan

**Keywords:** Biophysics, Cancer, Molecular medicine, Nanoscience and technology

## Abstract

We compared the effects of trivalent polyamines, spermidine (SPD) and norspermidine (NSPD), a chemical homologue of SPD, on the structure of DNA and gene expression. The chemical structures of SPD and NSPD are different only with the number of methylene groups between amine groups, [N-3-N-4-N] and [N-3-N-3-N], respectively. SPD plays vital roles in cell function and survival, including in mammals. On the other hand, NSPD has antitumor activity and is found in some species of plants, bacteria and algae, but not in humans. We found that both polyamines exhibit biphasic effect; enhancement and inhibition on *in vitro* gene expression, where SPD shows definitely higher potency in enhancement but NSPD causes stronger inhibition. Based on the results of AFM (atomic force microscopy) observations together with single DNA measurements with fluorescence microscopy, it becomes clear that SPD tends to align DNA orientation, whereas NSPD induces shrinkage with a greater potency. The measurement of binding equilibrium by NMR indicates that NSPD shows 4–5 times higher affinity to DNA than SPD. Our theoretical study with Monte Carlo simulation provides the insights into the underlying mechanism of the specific effect of NSPD on DNA.

## Introduction

Polyamines are small polycationic organic molecules that are found in all living organisms^1–3^. Intracellular concentrations of polyamines reach millimolar levels^4^. It has been shown that polyamine synthesis increases in actively proliferating cells and tumor cells^5–7^. Polyamines are involved in many biological functions. For example, polyamines are important in cell proliferation and differentiation, apoptosis, protection from oxidative damage and gene regulation^8–14^. Because of their cationic nature, they can interact with negatively charged macromolecules such as DNA, RNA and proteins, thereby affecting the structure and function of these macromolecules. It is well known that polyamines induce DNA compaction/condensation^15–22^. Several *in vitro* studies have investigated the physical process of DNA packaging and the physicochemical and biological properties of compacted DNA^23–28^. Regarding the potential to induce DNA compaction, it is well known that the valence of polyamines is critical^29,30^. The geometric arrangement of amine groups as well as chirality are also important factors in the ability to induce DNA compaction^31^. Atomic force microscopy (AFM) studies have revealed different morphologies of DNA depending on polyamine concentration and structure^27,32,33^. The changes in DNA conformation caused by polyamines could be related to genetic activity^34–37^. Recently, it was found that polyamines exert biphasic effects, enhancement and inhibition, on gene expression depending on their concentrations^38^. AFM observations showed that the enhancement correlates with a flower-like conformation with parallel ordering of DNA segments. On the other hand, the complete inhibition is caused by folding transition onto a tightly packed DNA conformation.

In this study, we compared the effects of trivalent polyamines, spermidine (SPD) and norspermidine (NSPD), a structural homologue of SPD, on the structure of DNA and gene expression. NSPD has antitumor activity and is found in some species of plants, bacteria and algae, but not in humans^3,39–46^. Although it has been argued that such antitumor activity is due to competition with natural polyamines for cellular uptake and accumulation^41,47^, the difference in their direct effects on DNA is still unclear. We also performed a theoretical study using a Monte Carlo simulation on the underlying mechanism of the different nature of interaction between SPD and NSPD on double-stranded DNA.

## Experimental Results

### Activity of gene expression

**Figure 1:**
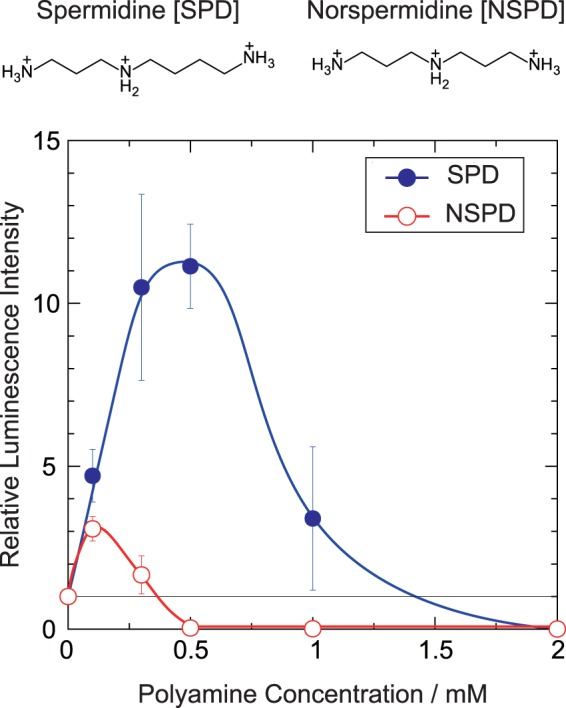
Efficiency of gene expression depending on the concentrations of SPD and NSPD. The longitudinal axis is the relative emission intensity of the luciferin-luciferase reaction. The DNA concentration was fixed at 0.3 μM.

The effects of SPD and NSPD on the activity of gene expression were evaluated through an *in vitro* cell-free luciferase assay. Figure 1 shows the relative luminescence intensity as a marker of gene expression activity at various polyamine concentrations, where the intensity is normalized to the control (=1), i.e., in the absence of polyamine. The results showed that both polyamines have a biphasic effect, enhancement and inhibition, on gene expression depending on their concentrations. The gene activity increased with an increase in the SPD concentration and reached a maximum at around 0.3 mM. As the SPD concentration increased further, the activity gradually decreased and was completely inhibited above 2 mM of SPD. Biphasic effect was also observed with NSPD: the activity reached a maximum at 0.1 mM and complete inhibition was observed above 0.5 mM. It is noted that the maximum level of gene activity with SPD was significantly higher, ca. 5-fold, than that with NSPD.

### Higher-order structure of DNA

**Figure 2:**
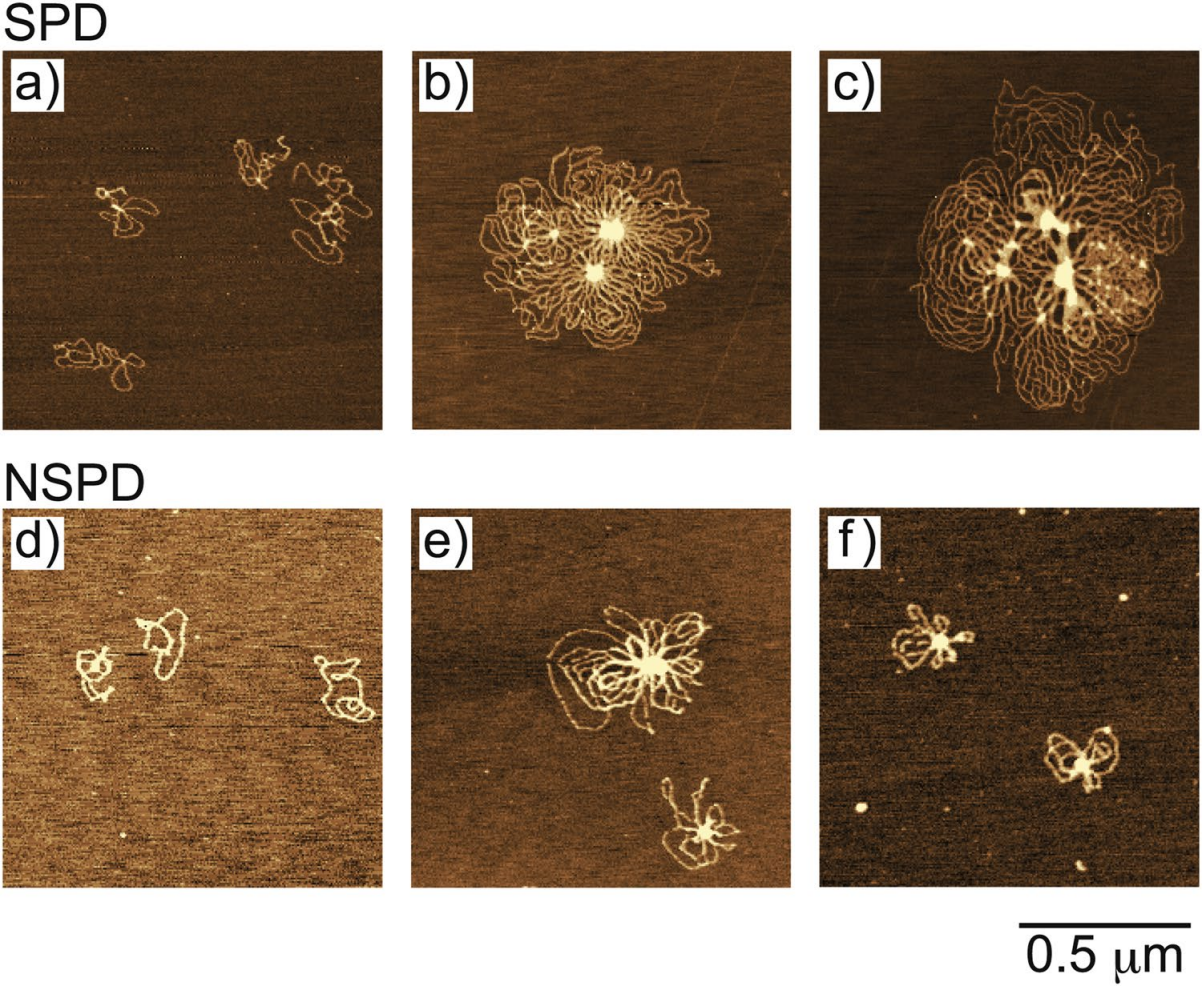
AFM images of plasmid DNA (4331 bp) in the presence of SPD and NSPD. Polyamine concentrations were (**a**,**d**) 0.1 mM, (**b**,**e**) 0.5 mM, and (**c**,**f**) 1 mM. The DNA concentration was 0.3 μM in nucleotide units.

To clarify whether the above difference in gene expression is due to the effects of polyamines on the higher-order structure of DNA, we investigated polyamine-induced changes in the structure of DNA by AFM and fluorescence microscopy (FM). Figure 2 shows typical AFM images of plasmid DNA (4331 bp) in the presence of SPD or NSPD. At 0.1 mM SPD or NSPD, DNA molecules were randomly dispersed (Fig. 2a,d). With an increase in the SPD concentration, a self-assembled flower-like structure appeared, where multiple loops crossed to form multiple nodal points (Fig. 2b). As the SPD concentration further increased, multiple DNA molecules formed a larger flower-like structure (Fig. 2c). Similar AFM observations of a flower-like structure with SPD were reported by Fan and Hoh^48^. On the other hand, in the presence of NSPD, the multimolecular loops tended to cross at a single point (Fig. 2e and f) and formed a smaller flower-like structure. These AFM observations imply that the difference by only one methylene group between SPD and NSPD leads DNA molecules to exhibit different conformations.

**Figure 3:**
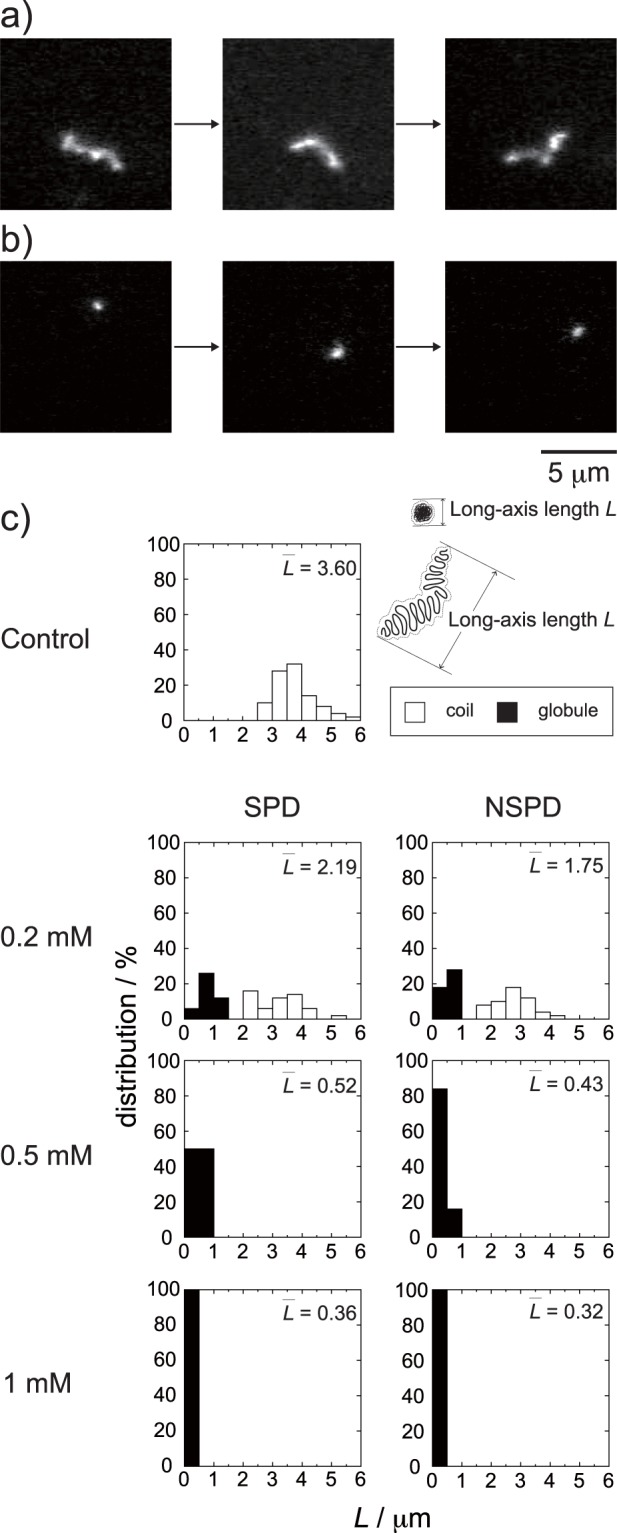
Typical FM image of a single T4 DNA molecule undergoing the Brownian motion in solution; (**a**) in the absence and, (**b**) in the presence of 1 mM NSPD. The time difference between neighbouring frames is 1 s. (**c**) Distribution of the long-axis length *L* of T4 DNA in solution at different concentrations of SPD and NSPD. The DNA concentration was fixed at 0.1 μM in nucleotide units. $\bar{L}$ (μm) is the ensemble average for 50 DNA molecules.

Next, we performed direct real-time monitoring of polyamine-induced changes in the conformation of DNA in aqueous solution by FM. Figure 3a,b shows representative FM images of thermally fluctuating T4 DNA molecules in the absence or presence of NSPD. Individual DNA molecules were observed as an elongated random coil and exhibited translational and intramolecular Brownian motion in aqueous solution (Fig. 3a). Upon the addition of NSPD to the DNA solution, individual DNA molecules exhibited a structural transition from a coil state to a compact globule state (Fig. 3b). Figure 3c shows histograms of the long-axis length *L* of DNA as a function of the concentration of polyamines together with an assignment of the conformational characteristics in FM images. Both NSPD and SPD caused shrinkage of DNA molecules with an increase in their concentrations. The potency for inducing DNA shrinkage was slightly greater for NSPD.

We have also examined the effect of SPD and NSPD on the secondary structure of DNA through CD measurements and found that the secondary structure retained the B form for both polyamines under the present conditions. (See Fig. S1 in the Supplementary Information).

### Evaluation of binding equilibrium by ^1^H NMR

**Figure 4:**
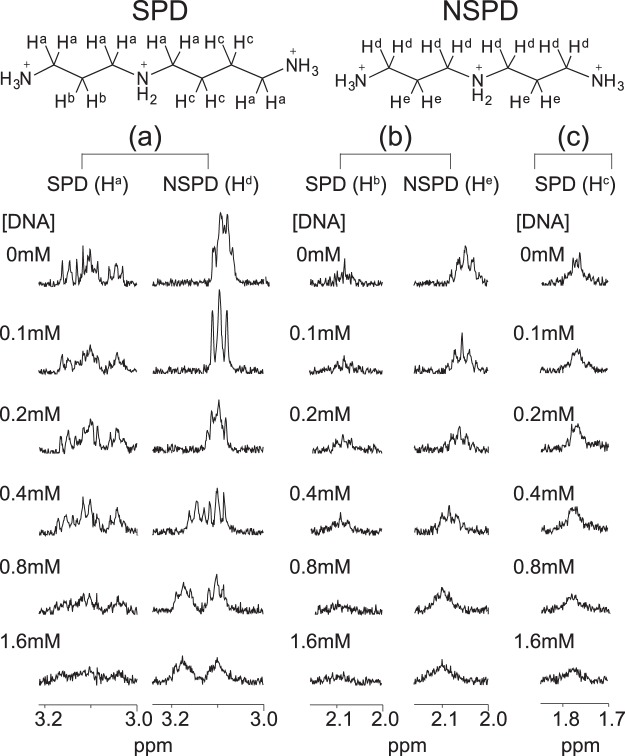
^1^H NMR spectra for methylene groups of SPD and NSPD at different concentrations of CT DNA. The polyamine concentration was fixed at 0.1 mM. (**a**) δ = 3.20–3.00 ppm, (**b**) 2.15–2.00 ppm, and (**c**) 1.85–1.70 ppm.

To evaluate the binding affinity of these polyamines for DNA, we performed ^1^H NMR titration experiments using CT DNA. Figure 4 shows the ^1^H NMR signals of SPD and NSPD in 10 mM Tris-DCl buffer (pD 7.5). The spectrum of SPD was composed of three resonances at around δ = 3.20–3.00, 2.15–2.00 and 1.85–1.70 ppm, and the spectrum of NSPD was composed of two at around δ = 3.20–3.00 and 2.15–2.00 ppm. Peak assignments are summarized in Fig. 4. DNA signals were not observed in the ^1^H NMR spectra of mixtures of the polyamines and DNA because of the significant decrease in *T*_2_, transverse relaxation time, for the protons on the high-molecular-weight CT DNA^49^. Upon addition of CT DNA, the intensity of the signals observed for SPD and NSPD generally decreased as the DNA concentration increased. This trend indicates that the DNA-bound fraction of polyamines is not detectable in the ^1^H NMR spectrum. In the case of SPD, the chemical shift and the widths of the signals remained essentially constant, similar to our previous results with chiral spermine analogs^31^.

**Figure 5:**
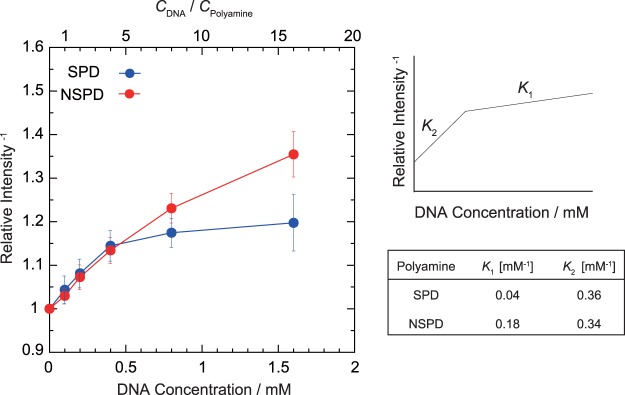
Left: Change in the intensity of ^1^H NMR signals depending on the concentration of CT DNA. The longitudinal axis is the inverse of the ^1^H NMR intensity evaluated through the integration of the observed signals, where all of the ^1^H signals are well-separated as shown in Fig. 4. The signal intensities in the graph were calculated based on the sum of all of the integrated values for the spectra of SPD and NSPD, so as to minimize the experimental error. Right: Schematic illustration showing how to evaluate the binding equilibrium constant, *K*, from the slope of the graph.

Based on the experimental data on the change in ^1^H NMR signal intensity, we evaluated the binding equilibrium constant of polyamines to DNA. The longitudinal axis in Fig. 5 shows the inverse of the ^1^H NMR intensity evaluated by integration of the observed signals. All of the ^1^H signals are well-separated, as shown in Fig. 4. Thus, the signal intensities in Fig. 5 were calculated based on the summation of all of the integrated values for the spectra of SPD and NSPD. The right panel in Fig. 5 also shows each binding equilibrium constant, *K*, evaluated from the slope of the graph. At a DNA concentration lower than 0.5 mM, the binding constants *K*_2_ of SPD and NSPD are 0.36 and 0.34 mM^−1^, respectively, which are almost identical. At a DNA concentration higher than 0.5 mM, however, the binding constants *K*_1_ of SPD and NSPD differed, and were calculated to be 0.04 and 0.18 mM^−1^, respectively. This result indicates that NSPD binds to DNA more strongly than SPD at a higher DNA concentration, i.e., under the condition that the polyamine concentration is low with respect to that of DNA.

### Summary of experimental observations

We found that the biphasic effects, enhancement and inhibition on gene expression, are induced by both polyamines (Fig. 1). Moreover, it was revealed that SPD significantly promotes gene expression, while NSPD has a weaker effect (Fig. 1). AFM observations indicated clear differences between SPD and NSPD on the morphology of DNA; SPD tends to induce a larger flower-like structure than NSPD (Fig. 2), suggesting that DNA segments tend to align in a parallel manner. FM observations demonstrated that NSPD induces shrinkage/compaction with a higher potency than SPD (Fig. 3). This trend in the effect on the DNA conformation corresponds to the inhibitory effect on gene expression; NSPD suppresses gene expression at a lower concentration than SPD (Fig. 1). In addition, through NMR measurements, it became clear that the binding constant of NSPD to DNA is evidently larger than that of SPD under the condition that the polyamine concentration is relatively low (Fig. 5).

## Discussion

**Figure 6:**
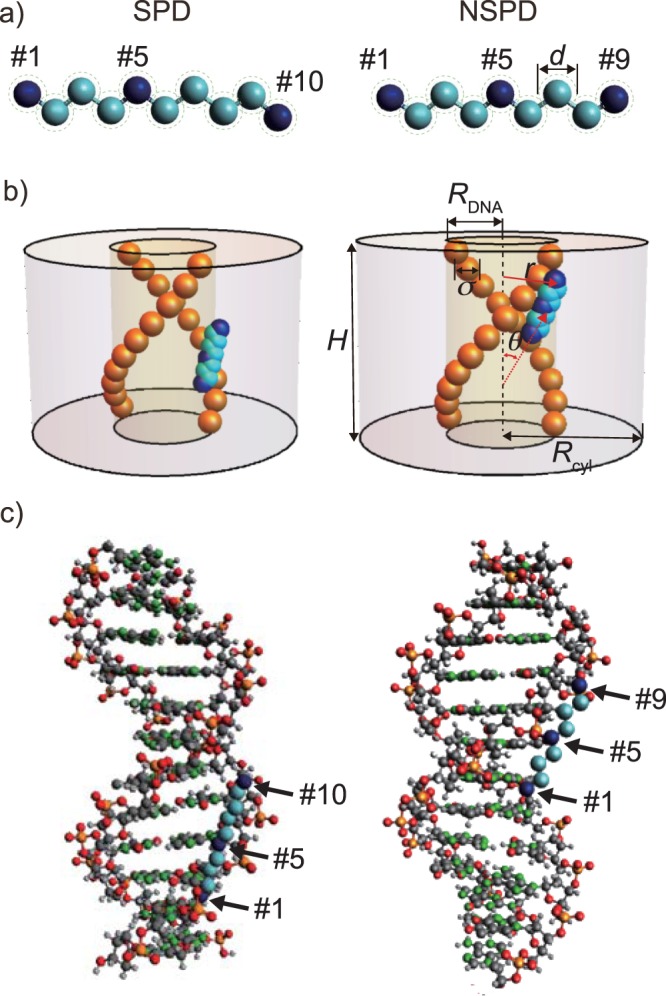
Simulation model for rigid SPD and NSPD in (**a**) and typical snapshots in the simulation for SPD and NSPD interacting with double-stranded DNA in (**b**) and (**c**), when the strength of electrostatic interaction *Γ* = 1.36 nm. For the numerical simulation in (**b**), pairs of charged spheres to mimic phosphate groups are placed in a soft cylinder of length *H* (=3.4 nm) and radius *R*_DNA_ (=1.0 nm) under a periodic boundary condition. The pictures in (**c**) represent the atomistic detail of the coarse-grained model. Hydrogen atoms are omitted for clarity. The positions of the three ammoniums in SPD and NSPD are indicated by #. In our model, a polyamine is allowed to partially penetrate into the DNA interior, which mimics minor and major grooves. The adjacent pairs of negatively charged moieties in the DNA model are separated by 0.34 nm.

In order to capture the essential feature on the manner of interaction of SPD and NSPD with DNA, we have adapted rather simple modelling for both polyamines and DNA. SPD and NSPD are represented as linear beaded chains as shown in Fig. 6a. Figure 6b shows a typical snapshot of NSPD and SPD interacting with a DNA segment, respectively, for electrostatic interaction of strength *Γ* = 1.36 nm (between an ammonium and a phosphate), together with the geometrical parameters defined in the present modeling for the coarse-grained model used in our numerical calculation. Note that the Mote Carlo simulation was carried out at T = 298 K to incorporate the effect of thermal fluctuation into the simulation. Detailed definition of *Γ* is given in Equation (S1) of the Supplementary Information. For NSPD, the model has 9 monomers, and the three ammonium groups are placed in positions #1, #5, and #9 (see e.g. the right panel in Fig. 6a). In contrast, for SPD, a total of 10 monomers are connected linearly, and the ammonium groups are labeled as #1, #5, and #10 based on their position in the chain molecule (the left panel in Fig. 6a). The details of numerical modeling together with parametrization are summarized in the Supplementary Information. Figure 6c shows schematics corresponding to the atomistic model. Both NSPD and SPD distribute close to the soft DNA boundary and polyamines tend to tilt by an angle of roughly 45° relative to the DNA axis. Inspection of both the detailed atomistic and coarse-grained models suggests that, for NSPD, the three ammoniums near the DNA soft boundary point in the same direction, which would induce a strong electrostatic attraction between NSPD and DNA. In contrast, for SPD, two ammoniums (#1 and #5) are distributed around the soft DNA boundary facing the phosphate groups, but due to its steric structure, the #10 ammonium points in a direction different from the other two ammoniums, and is located outside of the soft DNA boundary, which should weaken the interaction between SPD and DNA.

**Figure 7:**
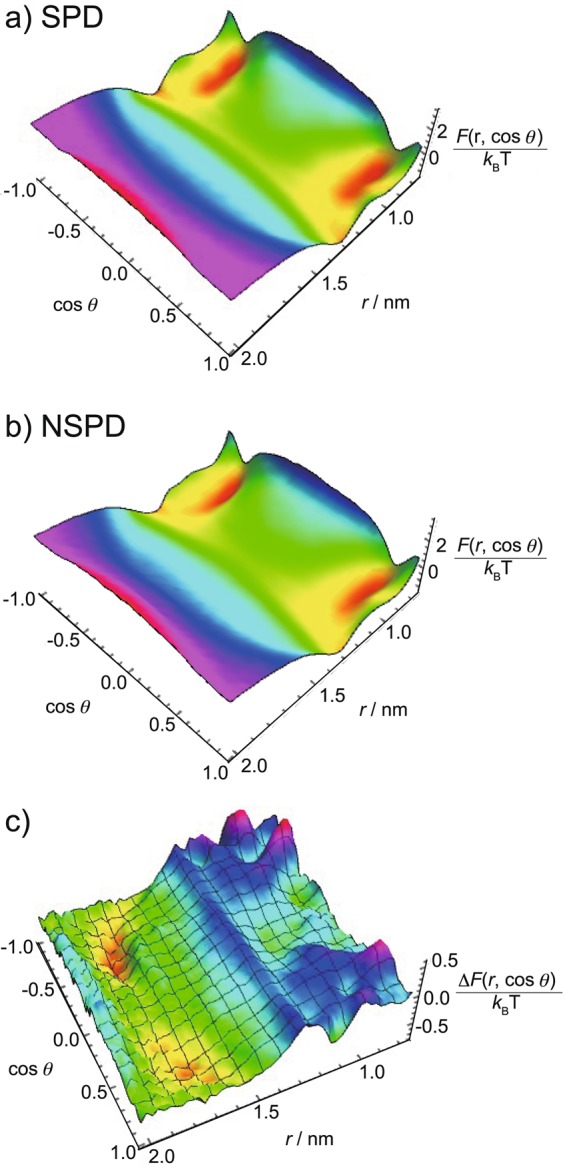
Plot of the free energy landscape *F*(*r*, cos *θ*)/*k*_B_*T* of SPD and NSPD when *Γ* = 1.36 nm and *R*_cell_ = 2.59 nm in (**a**) and (**b**), respectively, and the free energy difference between SPD and NSPD: Δ*F*(*r*, cos *θ*)/*k*_B_*T* = *F*_SPD_(*r*, cos *θ*) − *F*_NSPD_(*r*, cos *θ*) in (**c**).

To explore the manner of interaction of these polyamines with DNA in more detail, we calculate the two-variable density distribution function *ρ*_5_(*r*, cos *θ*); where the variables, *r* and *θ*, are the radial distance of the #5 ammonium and the angle of the vector measured from the #1 to #5 ammonium groups, respectively (cf. Equation (S3) in the Supplementary Information and Fig. 6). We then transform *ρ*_5_(*r*, cos *θ*) into the conditional free energy *F*(*r*, cos *θ*)/*k*_B_*T* = −ln [*ρ*_5_(*r*, cos *θ*)]. The two-variable profile of the free energy provides visual insights into the differences between the translational and rotational degrees of freedom of a trivalent polyamine around a DNA. Figure 7a,b plot the free energy landscape of *F*(*r*, cos *θ*)/*k*_B_*T* for SPD and NSPD, respectively, when *Γ* = 1.36 nm. The polyamines show similar profiles in the plot of *F*(*r*, cos *θ*)/*k*_B_*T*. First, in the area where the radial distance of the #5 ammonium *r* is around 1.3 nm or less, two distinct regions can be seen: (i) red and yellow regions, corresponding to lower-energy configurations, and (ii) green and blue regions, corresponding to higher-energy configurations. The lower free-energy area occurs roughly at |cos *θ*| between 0.5 and 1, and the higher free-energy area is approximated at |cos *θ*| between 0 and 0.5. Namely, both SPD and NSPD display a preferential orientation, and the lowest free-energy configuration is found at the red-color region with *θ* = ±40–50° (similar to the snapshot of SPD and NSPD in Fig. 6). Secondly, for *r* > 1.3 nm, the free energy increases steadily as *r* is increased. The detailed landscape also suggests that, at and beyond *r* = ~1.3 nm, both SPD and NSPD start to exhibit similar preference in all orientations, i.e., polyamines experience full rotation degrees of freedom. This is indeed an entropically favorable process. At *r* = ~1.3 nm, the polyamine may retain significant electrostatic attraction to DNA and gain enough rotational entropy simultaneously. Based on the landscape of *F*(*r*, cos *θ*)/*k*_B_*T*, it becomes possible to distinguish the strong binding regime from the weak binding regime at *r* near 1.3 nm. In contrast to SPD, the area of lower energy configurations (red and yellow regions) of NSPD is more expanded and the free energy landscape can be deeper.

To elucidate the quantitative difference between NSPD and SPD, we compute the conditional free energy difference: Δ*F*(*r*, cos *θ*)/*k*_B_*T* = *F*_SPD_(*r*, cos *θ*) − *F*_NSPD_(*r*, cos *θ*). Figure 7c plots the profile of Δ*F*(*r*, cos *θ*)/*k*_B_*T* for *Γ* = 1.36 nm and *R*_cyl_ = 2.59 nm. The hills in the landscape reflect configurations for which SPD has a higher free energy than NSPD, whereas in the valleys, SPD has a lower free energy than NSPD. The landscape is not totally symmetric at cos *θ* = 0 and *π* because SPD has an extra methylene group and is basically asymmetric. The hills emerge near *r* = 1 nm within a narrow range of cos *θ*, indicating that NSPD is preferentially located at around and within the soft DNA boundary with a specifically narrow range of preferential angles. This finding is consistent with the snapshots in Fig. 6 in which the three ammoniums in NSPD can orient themselves to face towards the charged phosphate groups. Moreover, the valleys in the free energy difference landscape are found to be away from the soft DNA surface at around *r* = 1.7–1.8 nm, a sign that SPD, in contrast to NSPD, tends to distance itself from the DNA surface. From these findings, it becomes evident that the extra methylene in SPD may weaken its electrostatic attraction with DNA, but enhances the spatial (translational and rotational) entropy of SPD around a DNA segment.

Our simulation model consists of one polyamine and one DNA segment, which corresponds to an experimental condition with lower concentrations of polyamines with respect to the phosphate groups in DNA; i.e., the line marked as *K*_1_ on the right side of the schematic plot in Fig. 5. To compute the binding constant corresponding to *K*_1_ deduced from the NMR measurement, in the simulation we monitor those binding events exclusively when all of the three ammoniums in polyamine simultaneously bind to the phosphates in DNA. To account for binding, the distance between the central nitrogen atom of an ammonium ion and the central phosphorous atom in a phosphate ion needs to be defined to differentiate binding from non-binding for these two types of ions. If we take the radii of ammonium and phosphate as 0.137 nm and 0.258 nm^50,51^, respectively, as well as the hydrogen bonding length (N-H-O-P) between the hydrogen in ammonium and the oxygen in phosphate as 0.174 nm^52^, the maximum length between an ammonium ion and a phosphate ion under the binding state can be estimated to be 0.569 nm. Note that 0.258 nm is actually the value for sulfate ion, which is considered to be similar to a phosphate ion in actual atomistic modeling^51^. By considering the angle of hydrogen bonding between ammonium and phosphate, the length between an ammonium ion and a phosphate ion (NH-O-P) may vary from 0.404 nm, 0.453 nm to 0569 nm (corresponding to 90°, 105° to 180°). In this work, we assume that a length 0.453 nm is the maximum length that allows binding between an ammonium and a phosphate. Thus, we can now calculate the binding constant *K*_*i*_ between polyamine and phosphate as follows:

1 $${K}_{i}=[Polyamine-Phosphate]/[Polyamine][Phosphate]\,$$

where *i* denotes SPD or NSPD. [*Polyamine*], [*Phosphate*], and [*Polyamine-Phosphate*] are the concentrations of free polyamine, free phosphate, and binding between them, respectively. The concentration ratio, [*Polyamine-Phosphate*]/[*Phosphate*], is determined from the bound and free polyamine as in Eq. (2).

2 $$[Polyamine-Phosphate]/[Polyamine]=\,(N(binding)/{V}_{b})/(N(nonbinding)/{V}_{f})\,$$

where *N*(*binding*) and *N*(*nonbinding*) are the average probability of a polyamine bound to and free from DNA, respectively, and *N*(*binding*) + *N*(*nonbinding*) = 1. *N*(*binding*) is calculated from the fraction of the events in which all of the three ammonium groups of polyamine simultaneously bind to DNA during the simulation. Thus, *N*(*nonbinding*) includes the other fractions of which any of the ammonium group(s) in a polyamine are free from binding to the phosphate group. Further details about *N*(*binding*) are given in Supplementary Information. *R*_cut_ is chosen to be 1.3 nm. *V*_b_ and *V*_f_ denote the volumes where bound and free polyamines are located, respectively, and are estimated based on the free energy diagrams in Fig. 7 by adapting the following Eqs. (3) and (4) with *μ* = cos *θ*:

3 $${V}_{b}=[2\pi H{\int }_{{R}_{{\rm{cut}}}}^{{R}_{{\rm{DNA}}}-\tfrac{\sigma }{2}}rdr][2{\int }_{0.5}^{1}d\mu {\int }_{0}^{2\pi }d\varphi ]$$

4 $${V}_{f}=[2\pi H{\int }_{{R}_{{\rm{cut}}}}^{{R}_{{\rm{DNA}}}-\tfrac{\sigma }{2}}rdr][2{\int }_{0}^{0.5}d\mu {\int }_{0}^{2\pi }d\varphi ]+4\pi H{\int }_{{R}_{{\rm{cut}}}}^{{R}_{{\rm{cyl}}}}rdr$$

Since our investigation is directed to a low polyamine-to-phosphate concentration ratio, we approximate the free phosphate concentration [*Phosphate*] in Eq. (1) to be the same as the overall phosphate concentration in the simulation. Note that when the three ammoniums bind to DNA phosphates, the orientation of the polyamine falls in the range of 40–80 degrees that coincide with the free energy minimum valleys in Fig. 7a,b.

**Figure 8:**
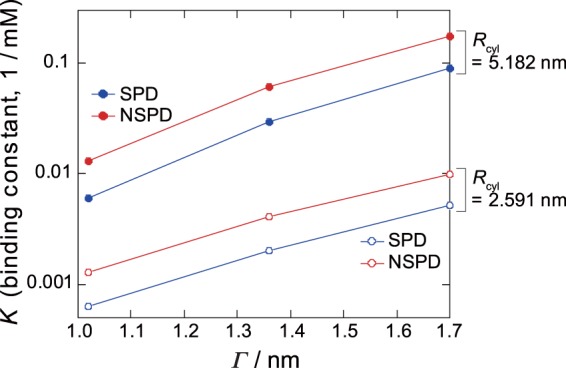
Plot of the binding constants *K* for both NSPD and SPD with DNA as a function of *Γ* when *R*_cyl_ = 5.182 and 2.591 nm. *Γ* is a parameter to represent the strength of Columbic interaction (see Supplementary Information).

Figure 8 plots the calculated binding constant *K* for NSPD and SPD as a function of *Γ* for two different sizes of cylindrical simulation cell with *R*_cyl_ = 5.182 and 2.591 nm. Here, it is noted that *K*_NSPD_ is always greater than *K*_SPD_ for any given *Γ* and *R*_cyl_. The binding constant ratio *K*_NSPD_/*K*_SPD_ on the data in Fig. 8 is around 2, being slightly smaller than the experimental estimation (around 4). This small difference is attributable to the neglect of the contribution from translational entropy of counter ions^20,29^ and from the hydration effect including the ionization balance of the free ammonium groups. Nevertheless, it is regarded that the simulation reproduces the essentials on the difference of binding nature between SPD and NSPD in our experiments. The weaker binding of SPD implies a larger surviving negative charge on a double-stranded DNA, which caused larger repulsive interaction between DNA segments. This effect may promote the parallel alignment of DNA chains, as indicated in our AFM observations in Fig. 2, i.e., generation of flower like structure with SPD. It has been suggested that self-avoiding effect of DNA segments in a lightly shrunken DNA chain promote parallel ordering, under the similar mechanism of stabilization with liquid crystalline ordering^38^.

Summarizing the above mentioned numerical study, it has become clear that NSPD has preferential configurations in which NSPD binds strongly with DNA, but SPD gains translational and rotational degrees of freedom (entropy-favored) while it is located away from DNA. In other words, SPD plays an essential role in reducing electrostatic attraction between polyamine and DNA (by decreasing the linear charge density of a polyamine). As the result, SPD exhibits weaker binding with DNA than does NSPD. By analyzing the state in which three ammoniums bind simultaneously to DNA phosphates, the binding constant of polyamines with DNA was estimated. The results revealed that the binding constant ratio between NSPD and SPD deduced theoretically is consistent with the trend observed in the NMR experiment. Such scheme of the specific feature of the interaction between DNA and polyamines will provide a useful insight for the development of both fundamental and application studies on polyamines^15–22,53–58^.

## Conclusions

Both experimental and theoretical results clearly show that the difference by only one methylene group between SPD and NSPD causes marked differences in the binding affinity and DNA structure, which leads to significant differences in the gene expression profile *in vitro*. This means that the replacement of SPD by NSPD results in a significant loss of genetic activity; NSPD induces weaker enhancement at low concentrations and stronger inhibition at high concentrations. These findings shed light on the underlying mechanism why SPD and NSPD exhibit markedly different activity *in vivo*. For example, Porter and Bergeron reported that NSPD causes a loss of biological activity in cultured L1210 leukemia cells^47^. Silvia *et al*. also evaluated the cytotoxic effects of NSPD and its Pd(II) complex on human breast cancer cell lines and demonstrated that these compounds cause growth inhibition and cell death^46^.

From a methodological point of view, combinatorial study with structural and functional analysis as adapted in the present study would be useful to obtain the essential features of the ligand-DNA interaction. Studies along such direction are expected to contribute for the design of novel polyamines as the candidate of potent antitumor active drug^61^.

## Materials and Methods

### Materials

Spermidine trihydrochloride (SPD) was purchased from Nacalai Tesque (Kyoto, Japan). Bis(3-aminopropyl)amine (NSPD) was purchased from Sigma-Aldrich (St. Louis, MO, USA). The fluorescent cyanine dye YOYO-1 (1,10-(4,4,8,8-tetramethyl-4,8-diazaundecamethylene)bis[4-[(3-methylbenzo-1,3-oxazol-2-yl)methylidene]-l,4-dihydroquinolinium] tetraiodide) was purchased from Molecular Probes, Inc. (Eugene, OR, USA). The antioxidant 2-mercaptoethanol (2-ME) and calf thymus DNA (CT DNA: 8–15 kbp) were purchased from Wako Pure Chemical Industries (Osaka, Japan). T4 GT7 phage DNA (contour length: 57 μm, 166 kbp) was purchased from Nippon Gene (Toyama, Japan). Plasmid DNA (Luciferase T7 Control DNA, 4331 bp) containing a T7 RNA polymerase promotor sequence was purchased from Promega (Madison, WI, USA). Other chemicals obtained from commercial sources were of analytical grade.

### Luciferase assay for gene expression

A cell-free luciferase assay with a TnT T7 Quick Coupled Transcription/Translation System (Promega) was carried out according to the manufacturer’s instructions as follows. Plasmid DNA with the promotor region of T7 RNA polymerase was used as the DNA template. The DNA concentration was 0.3 μM in nucleotide units. The reaction mixture was incubated for 90 min at 30 °C on a Dry Thermo Unit (TAITEC, Saitama, Japan). Luciferase expression was evaluated following the addition of luciferase substrate (Luciferase Assay Reagent, Promega) by detecting the emission around 565 nm using a luminometer (MICROTEC Co., Chiba, Japan)

### AFM observations

For AFM imaging using an SPM-9700 (Shimadzu, Kyoto, Japan), 0.3 μM plasmid DNA (4331 bp) was dissolved in 10 mM Tris-HCl buffer solution at pH 7.5 with various concentrations of polyamines. The DNA solution was incubated for more than 10 min and then transferred onto a freshly cleaved mica surface. Subsequently, the sample was rinsed with ultra-pure water, dried with nitrogen gas and imaged by AFM. All measurements were performed in air using the tapping mode. The cantilever, OMCL-AC200TS-C2 (Olympus, Tokyo, Japan), was 200 μm long with a spring constant of 9–20 N/m. The scanning rate was 0.4 Hz and images were captured using the height mode in a 512 × 512 pixel format. The obtained images were plane-fitted and flattened by the computer program supplied with the imaging module.

### FM observations

To visualize individual DNA molecules in solution by FM, a large DNA, T4 GT7 DNA was used. DNA was dissolved in a 10 mM Tris-HCl buffer and 4% (v/v) 2-ME at pH 7.5 in the presence of various concentrations of polyamines (0–1000 μM). Measurements were conducted at a low DNA concentration (0.1 μM in nucleotide units). YOYO-1 (0.05 μM) was added to the DNA solution and single-molecule observations were performed with an inverted fluorescence microscope (Axiovert 135, Carl Zeiss, Oberkochen, Germany) equipped with a 100× oil-immersion objective lens and fluorescent illumination from a mercury lamp (100 W) via a filter set (Zeiss-10, excitation BP 450–490; beam splitter FT 510; emission BP 515-565). Images were recorded onto a DVD at 30 frames per second with a high-sensitivity EBCCD (Electron Bombarded Charge-Coupled Device) camera (Hamamatsu Photonics, Shizuoka, Japan) and analyzed with the image-processing software ImageJ (National Institute of Mental Health, MD, USA). Based on the observation of time-successive images, the distribution of the long-axis length of DNA in solution was evaluated, and 50 DNA molecules were measured at each experimental condition.

### NMR titration experiments

The binding abilities of NSPD and SPD to CT DNA were investigated via ^1^H-NMR titration experiments by using a JEOL JNM-ECZ500R. All experiments were carried out in 10 mM Tris-DCl buffer (pD 7.5) and 3-(trimethylsilyl)-2,2′,3,3′-tetradeuteropropionic acid (TMSP-d_4_) was used as an internal reference. A presaturation technique was used for suppression of a water peak. Both polyamine samples were prepared at a concentration of 100 μM in 10 mM Tris-DCl buffer (pD 7.5). The CT DNA titrant sample (stock solution) was prepared at a concentration of 20 mM in D_2_O. Polyamine sample (0.6 mL) was introduced into the NMR tube, and increasing amounts of the titrant DNA solution were added. A progressive disappearance of the proton signals of the polyamine was observed, indicating a significant decrease of *T*_2_, lateral relaxation time of the ^1^H signals, caused by the binding of polyamines to CT DNA.

### Monte Carlo simulation

We conducted a Monte Carlo simulation with a simple model to elucidate the interaction between polyamine and DNA. Following the procedure in our previous work^22^, charged SPD and NSPD are both treated as rigid chain molecules, as shown in Fig. 6a, due to their large bending and torsional energies as well as high linear charge density. Such a rigid chain model is consistent with a recent atomistic simulation for multiple SPD molecules interacting with DNA^59^. The diameters of methylene and ammonium groups *d* are assumed to be 0.39 nm^60^, and each ammonium group carries +1 unit charge.

A model DNA segment consisting of 10 pairs of charged spheres to mimic phosphate groups is placed in a cylindrical simulation cell of length *H* = 3.4 nm and radius *R*_cyl_ as shown in Fig. 6b. The phosphate groups are modeled as charged spheres with −1 unit charge and diameter *σ* = 0.476 nm^50,51^. The two charged spheres of each pair are 180° apart around the DNA cylinder (of radius *R*_DNA_ = 1 nm). The electrostatic interactions are treated at the level of screened Coulomb potential. Further details of the model and Monte Carlo simulation are explained in the Supplementary Information.

## Supplementary information

Supplementary Information

## Data Availability

The datasets generated and/or analyzed during the current study are available from the corresponding authors on reasonable request.
