# Supplementary material for: Specific effects of antitumor active norspermidine on the structure and function of DNA
Source: Sci Rep. 2019 Oct 18;9:14971. doi: 10.1038/s41598-019-50943-1 (PMC6802174; doi:10.1038/s41598-019-50943-1)
Supplement: Supplementary file 1 — Supplementary Information [file 41598_2019_50943_MOESM1_ESM.pdf]

Supplementary Information

Specific effects of antitumor active norspermidine on the structure and function of DNA

Takashi Nishio[1], Yuko Yoshikawa[1], Chwen-Yang Shew[2,*], Naoki Umezawa[3], Tsunehiko Higuchi[3], Kenichi Yoshikawa[1,*]

[1] Faculty of Life and Medical Sciences, Doshisha University, Kyotanabe 610-0394, Japan
[2] Doctoral Program in Chemistry, The Graduate Center of the City University of New York, New York 10016, USA and Department of Chemistry, College of Staten Island, Staten Island, New York, 10314, USA
[3] Graduate School of Pharmaceutical Sciences, Nagoya City University, Nagoya 467-8603, Japan

Corresponding author: Kenichi Yoshikawa, Faculty of Life and Medical Sciences, Doshisha University, Kyoto 610-0321, Japan
TEL: +81-774-65-6243
FAX: +81-774-65-6243
e-mail: keyoshik@mail.doshisha.ac.jp

Correspondence may also be addressed to Chwen-Yang Shew.
Email: ChwenYang.Shew@csi.cuny.edu

1. CD measurements

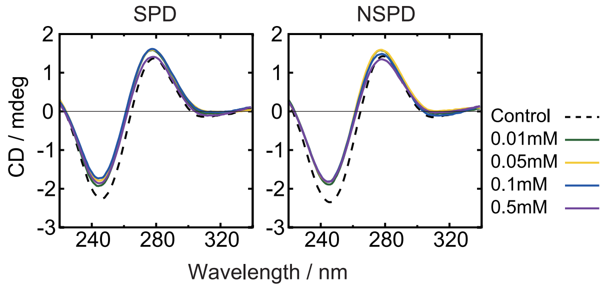

Figure S1: CD spectra of DNA at different concentrations of SPD and NSPD.

Figure S1 shows CD spectra of DNA with the addition of different concentrations of polyamines, where calf thymus (CT) DNA was adopted for the measurements. For both NSPD and SPD, there seems to be no apparent change in the CD spectra, indicating that the secondary structure retains the B-form under these conditions. CD spectra of CT DNA upon the addition of polyamines were measured at 25°C in 1 mM Tris-HCl buffer (pH 7.5) on a J-720W spectropolarimeter (JASCO, Tokyo, Japan). The DNA concentration was 30 μM in nucleotide units. Polyamine concentrations varied from 10 to 500 μM. The cell path length was 1 cm. Data were collected every 1 nm between 220 and 340 nm at a scan rate of 200 nm/min, and accumulated 3 times.

2. Original ^{1}H NMR spectra of the titration experiments for the data in Figure 4

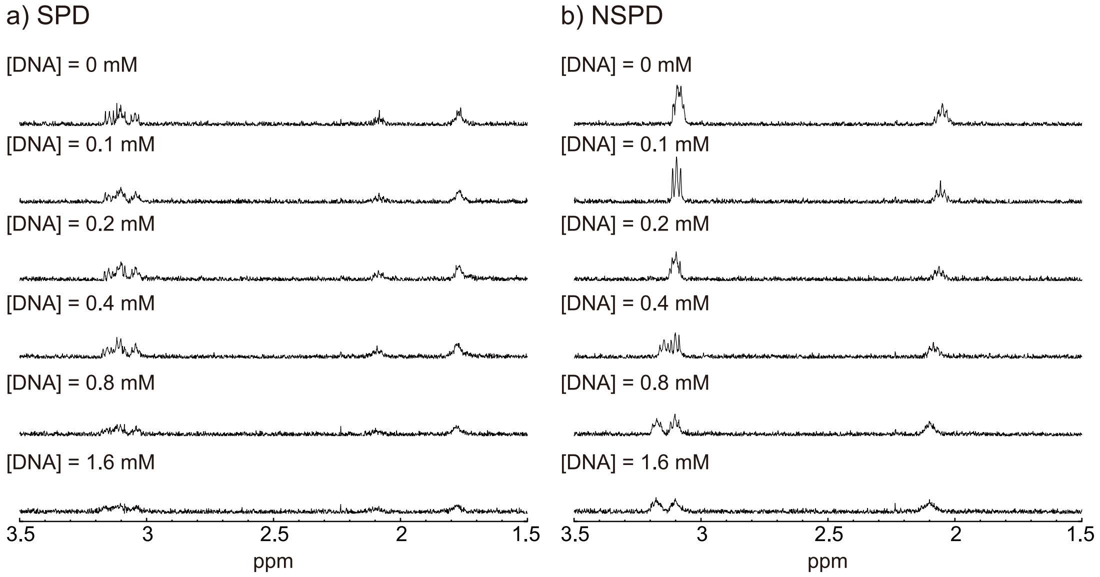

Figure S2: ^{1}H NMR spectra of SPD and NSPD at different concentrations of CT DNA. The polyamine concentration was fixed at 0.1 mM.

3. Details of numerical modeling

The SPD and NSPD conformations shown in Fig. 6 correspond to the lowest-energy conformer for each molecule, and can be obtained by using the energy minimization scheme of Avogadro software under UFF (Universal Force Field)[1]. To address the excluded volume of ammonium and methylene groups, each of these groups is modeled as a hard sphere of diameter $d = 0.39$ nm in Fig. 6a, similar to the coarse-grained polyethylene model[2]. Each of the three ammonium groups in SPD and NSPD is assigned +1 unit charge.

To model a DNA segment in the coarse-grained model, we adapt a charged DNA segment of one pitch of about 3.4 nm[3]. Figure 6 shows the model DNA segment consisting of 10 pairs of charged spheres (-1 unit charge for each sphere) around a soft cylinder of length $H = 3.4$ nm and radius $R_{\mathrm{DNA}} = 1$ nm. The charged spheres represent the phosphate groups of typical dimeter $\sigma = 0.476$ nm. The two charged spheres of each pair are 180° apart around the DNA cylinder ($R_{\mathrm{DNA}} = 1$ nm). The first pair starts at an axial location $z = 0.17$ nm above the bottom of the cylinder corresponding to the DNA segment ($z = 0$), and the remaining pairs are oriented 36°and elevated 0.34 nm sequentially. The final pair ends at a height 0.17 nm below the

top of the cylinder of the DNA segment (z = 3.4 nm).

In the investigated DNA model, 20 charged sites on the DNA segment are grouped into 10 pairs. The two charged spheres in each pair are separated by 180° around the cylinder and their separation is set at 2 nm, close to the width of a DNA molecule. These 10 pairs of charged groups are arranged from the bottom to the top of the cylinder by rotating 36° for each pair, and any two adjacent pairs are separated by 0.34 nm. The top and bottom pairs are 0.17 nm away from the ends of the cylindrical cell.

The above DNA segment will replicate itself in a one-dimensional periodic boundary condition along the axis of the DNA segment in a Monte Carlos simulation, and by implementing a one-dimensional Ewald summation[4], all the DNA images form an infinitely long DNA chain as in the traditional cell model[5]. Our simulation considers a rigid cylindrical cell with radius R_{cell} = 2.59 and 5.18 nm. The monomers (charged ammonium or neutral methylene) of a polyamine are not allowed to cross the rigid boundary of the simulation cell, and a monomer in the polyamine molecule can reach up to the radial position at $R_{\mathrm{cell}} - d/2$ (d: diameter of an ammonium or a methylene monomer). Nevertheless, monomers can penetrate the soft cylinder of the model DNA core (R_{DNA} = 1 nm) up to $R_{\mathrm{DNA}} - \sigma/2$ (σ: diameter of a coarse-grained phosphate group), similar to the empty space around the major and minor grooves of DNA that involve binding processes.

In our Monte Carlo simulation, we choose translation and rotation motion randomly with equal probability to sample configurations of the polyamine with temperature at T = 298 K. Also, the step sizes for the above two types of sampling schemes are adjusted to achieve an acceptance ratio around 50-60% under the Metropolis algorithm[6]. For each parameter set, a total of 9×10^8 moves are conducted in the simulation, and the first 10^8 moves are discarded to ensure convergence in calculations.

The electrostatic interaction between the monomers of a polyamine and a phosphate group is treated at the level of the primitive model with the screened Coulomb potential to incorporate water, counterions and coions implicitly into the interaction potential, given by

$$V(r)/k_BT = \infty \quad \text{if } r < r_c$$

$$= \Gamma q_i Q_j \exp(-\kappa r)/r \quad \text{if } r \geq r_c \qquad \text{(S1)}$$

where k_B is the Boltzmann constant; T is the temperature; r is the separation between a monomer in a polyamine and the coarse-grained phosphate group in the DNA; i is the i-th monomer in the polyamine with charge q_i (0 for a methylene group; + 1 for an ammonium group); j is the j-th phosphate group in DNA with charge Q_j ; Γ is the interaction strength; κ is the inverse Debye screening length; and r_c is approximated to be 0.31 nm, a typically closest distance between a charged amine and a phosphate group in DNA[7]. In this work, Γ ranges from 0.68 to 1.7 nm, about 1 to 2.4 Bjerrum lengths at 25°C to mimic the low dielectric regime around DNA with low water content, and the inverse Debye screening length κ is chosen to be 2.4 nm^{-1}. This inverse Debye screening length is set to reflect the low-water and low-electrolyte region close to the DNA surface.

In this work, we are particularly interested in the density distribution function of charged monomers (i.e., ammonium groups) in polyamine. We compute the reduced density distribution function of the i-th ammonium group $\rho_i(r)$ (defined in Fig. 6) as a function of its radial distance r measured from the center of the DNA axis for different Γ. To compute $\rho_i(r)$, the cylindrical simulation cell is divided into 100 layers along the radial direction, and then the average number of the charged monomer i in the k-th layer $B_k(r)$ (histogram) is calculated. $\rho_i(r)$ is obtained by

$$\rho_i(r) = V_{\text{cyl}}\, B_i(r)/v_i(r) \qquad \text{(S2)}$$

where $v_i(r)$ is the volume of the i-th layer, and V_{cyl} is the total volume of the cylindrical simulation cell to keep the density distribution function dimensionless. Moreover, the calculation of $\rho_i(r)$ is further extended

to the two-variable density distribution function $\rho_5(r, \cos\theta)$ for the ammonium group at the middle of the polyamine (#5 defined in Fig. 6) where θ is the angle between the direction of the DNA axis and the unit vector drawn from the ammonium at position #1 to that at position #5 (shown in Fig. 6), given by

$$\rho_5(r, \cos\theta) = V_{\mathrm{cyl}}\, B_5(r, \cos\theta)/(v_i(r)\Delta\mu) \quad \text{(S3)}$$

where $\mu = \cos\theta$ and $\Delta\mu$ is the interval of μ in the simulation. In the calculation, we divide the range of μ (between -1 and 1) into 50 intervals with $\Delta\mu = 0.04$, and the radial distance r (between $R_{\mathrm{DNA}} - \sigma/2$ and $R_{\mathrm{cell}} - d/2$) into 50 intervals with a total of 2500 intervals in the histogram $B_5(r, \cos\theta)$.

4. Additional explanation of the results of the numerical calculation

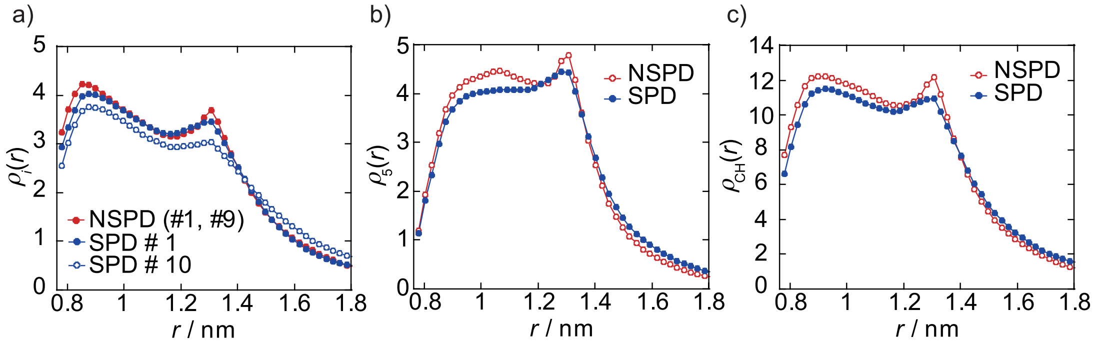

Figure S3: Plots of the (radial) density distribution function of the end ammonium groups in (a); of the middle ammonium group in (b); and of all three charged ammonium groups in (c) between NSPD, denoted by red symbols and lines, and SPD, denoted by blue symbols and lines, for $\Gamma = 1.36$ nm. The end ammoniums of SPD are located at #1 and #10, and those of NSPD are located at #1 and #9, as defined in Fig. 6. For both SPD and NSPD, the middle ammonium is positioned at #5.

Figure S3 compares the density distribution function of the end ammonium groups marked in Fig. S3a and the middle ammonium group in Fig. S3b between NSPD, denoted by red symbols and lines, and SPD, denoted by blue symbols and lines. Note that the end ammoniums of SPD are located at #1 and #10, and the end ammoniums of NSPD are located at #1 and #9, as defined in Fig. 6, for $\Gamma = 1.36$ nm. In Fig. S3a, all density distribution functions exhibit two peaks: one is located near r = 0.85 nm and the other is

positioned around $r = 1.3$ nm. Beyond $r = 1.3$ nm, all density distribution functions decrease monotonically, indicating that, beyond this distance, the electrostatic interaction decreases significantly due to a more marked separation between oppositely charged ammonium and phosphate. Also, Fig. S3a shows that the two end ammoniums (#1 and #9) in NSPD are identical because they are symmetric to each other against the central monomer (#5) in the chain molecule. In contrast, the two end ammoniums (#1 and #10) in SPD are asymmetric. The density distribution function of #1 ammonium ρ_1 in SPD has a similar magnitude as that of NSPD, and it becomes slightly lower than that of NSPD at smaller r. At the #10 ammonium of SPD, the density distribution function ρ_{10} is significantly lower at $r < 1.3$ nm due to the additional methylene group on the side of the #10 ammonium in SPD. This extra methylene likely reduces the local charge density as well as the electrostatic interaction between the #10 ammonium and the charged phosphates. In Fig. S3b, ρ_5 for the middle ammonium (#5) of SPD is significantly lower than that of NSPD below $r < 1.2$ nm, like ρ_{10} in SPD, and ρ_5 has a similar magnitude for both SPD and NSPD at around $r = 1.3$ nm, like ρ_1 in SPD. Namely, ρ_5 exhibits features in-between ρ_1 and ρ_{10} for SPD, and serves as a good physical quantity to differentiate the distinct behavior between SPD and NSPD. Figure S3c is the radial density distribution function of all ammonium groups: the density distribution of NSPS is greater than that of SPD below $r =$ 1.4 nm or so, but this trend reverses for $r > 1.4$ nm.

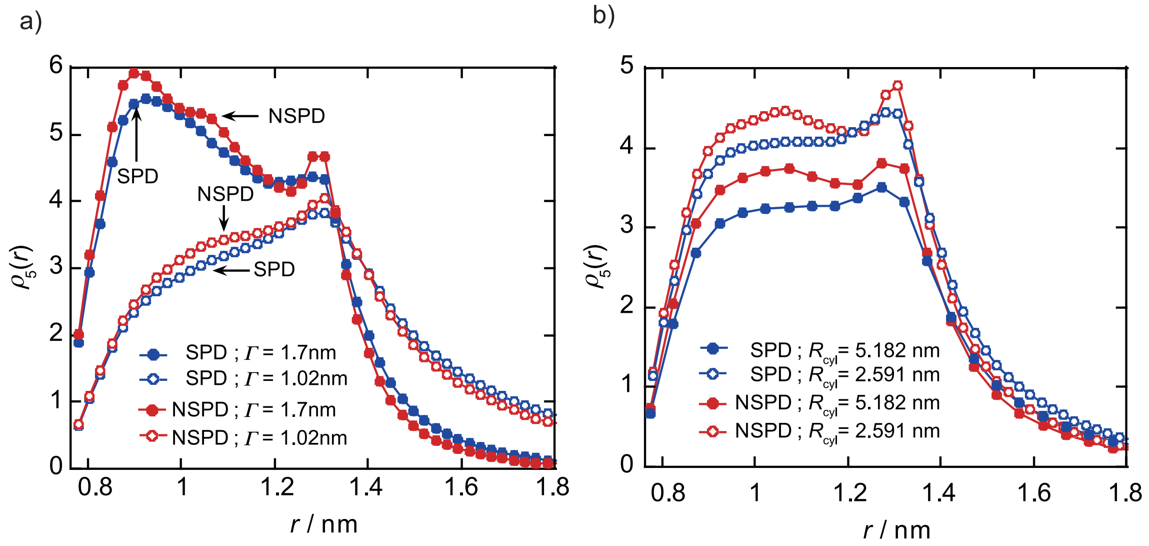

Figure S4: Comparison of the density distribution function of the middle ammonium group at position #5 (defined in Fig. 6) ρ_5(r) for both NSPD (red color) and SPD (blue color) for Γ = 1.02 nm, denoted by open symbols, and 1.7 nm, denoted by closed symbols in (a) as well as simulated ρ_5(r) for a cylindrical simulation cell of radius R_{cyl} = 5.182 (solid symbols and lines) and 2.591 nm (open symbols and lines) when Γ = 1.36 nm in (b).

Figure S4 plots ρ_5 for both SPD and NSPD, as marked, for Γ = 1.02 nm, denoted by open symbols, and for Γ = 1.7 nm, denoted by closed symbols. A greater Γ induces stronger electrostatic interaction between a charged polyamine and DNA. As a result, in the case of Γ = 1.7 nm, ρ_5 shows a greater density in the region inside the soft DNA boundary (r < 1 nm), whereas for Γ = 1.02 nm, ρ_5 shows that the middle ammonium distributes preferentially outside of the soft DNA boundary. For both Γ values, the ρ_5 of SPD tends to be smaller than that of NSPD should be at around r < 1.4 nm, suggesting that the electrostatic attraction between SPD and DNA is effectively weaker than the interaction between NSPD and DNA.

To further test the effect of electrostatic interaction, in Fig. S4, we plot the ρ_5 of both NSPD (red symbols and lines) and SPD (blue symbols and lines) for Γ in (Fig. S4a) with Γ = 1.02 and 1.7 nm, denoted by open and solid symbols, respectively, and for R_{cyl} = 2.591 and 5.182 nm, denoted by open and solid symbols, respectively, in (Fig. S4b). For weak electrostatic interaction Γ = 1.02 nm, both NSPD and SPD display a greater tendency to be away from the DNA surface, whereas under strong electrostatic interaction Γ = 1.7 nm, both NSPD and SPD are more likely to be near the DNA surface. For both Γ values, when r < 1.4 nm or so, the probability of finding NSPD near DNA is greater than that for SPD, but for r > 1.4 nm, we obverse

an opposite trend, in that SPD exhibits a greater probability density distribution than NSPD. This feature is basically similar to that in Fig. S3b. Note that the local peak near r = 1.3 nm is present for the density distribution function ρ_5 for both NSPD and SPD in Figs S3 and S4. The density distribution ρ_5 can clearly show a distinct difference due to electrostatic interaction between NSPD and SPD.

In Fig. S4b, upon doubling R_{cyl}, ρ_5 for NSPD and SPD show similar features, and the difference between NSPD and SPD follows the same trend as those in Figs S3 and S4a. For larger R_{cyl} (= 5.182 nm), ρ_5 of NSPD and SPD become smaller than those for smaller R_{cyl} (= 2.591 nm).

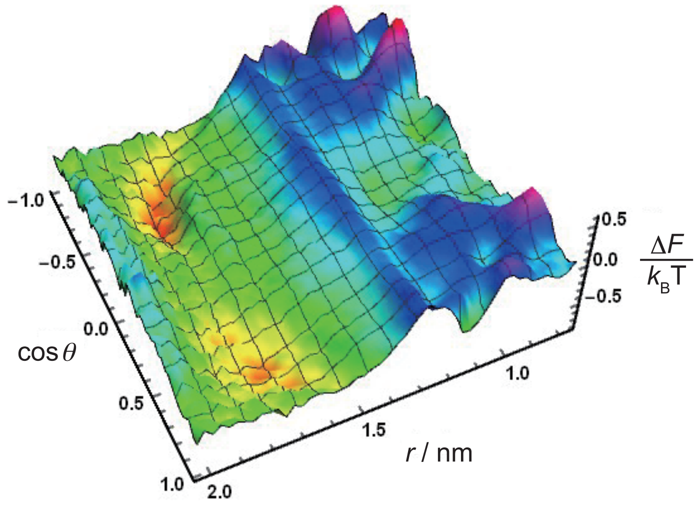

Figure S5: Plot of $\Delta F(r, \cos\theta)/k_BT$ (= $F_{SPD}(r, \cos\theta)$ - $F_{NSPD}(r, \cos\theta)$) for Γ = 1.7 nm. r is the radial distance of the middle ammonium at position #5 for SPD or NSPD and θ is the angle between the unit vector measured from the #1 to #5 ammonium and the DNA main axis. Both r and θ are defined in Fig. 6.

Figure S5 is the same plot as in Fig. 7c except that Γ is increased to 1.7 nm. The qualitative features are the same between Fig. 7c and S5, but for the greater Γ, the hills and valleys in the free energy difference landscape become more pronounced. Near 1.3 nm, a significant increase in probability is observed for different θ-angles due to rotational entropy, which increases rotational degrees of freedom. These features help us understand Fig. S4a, in which, for a greater Γ, $\rho_5(r)$ has a maximum near DNA due to the more energetically favorable process, but for a smaller Γ, $\rho_5(r)$ has a maximum away from DNA because polyamine gains entropy through rotation and translational motion along the direction of the DNA axis.

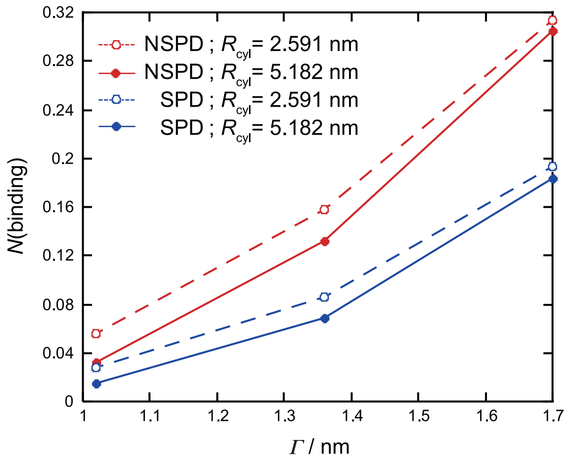

Figure S6: Plot of the simulated average number of SPD (denoted by blue circles) and NSPD (denoted by red circles) bound to DNA (N(*binding*)) for R_{cyl} = 2.591 nm (open symbols) and 5.182 nm (solid symbols).

Figure S6 compares the simulated average probability of a polyamine bound to DNA (N(*binding*)) as a function of Γ between NSPD (blue circles) and SPD (red circles) for R_{cyl} = 2.591 nm (open symbols) and 5.182 nm (solid symbols). First, a binding event is defined as the case when all of the three ammonium groups in polyamine simultaneously bind to the phosphate groups in DNA in simulation (when their distance falls below 0.453 nm, as discussed in the main text, but above the distance accounted for their excluded volume interaction). N(*binding*) is then calculated from B(*binding*)/B(*total*), where B(*binding*) is the number of the configurations with binding events and B(*total*) is the total number of configurations sampled in the simulation.

In general, N(*binding*) increases as Γ is increased due to stronger electrostatic attractions between polyamine and DNA. For a given R_{cyl}, the N(*binding*) of NSPD is greater than that of SPD for all Γ, consistent with the picture that NSPD interacts more strongly with DNA. For a greater R_{cyl}, N(*binding*) tends to be smaller for a given type of polyamine due to competition from configurational entropy. But for the very high Γ(= 1.7 nm), the effect of R_{cyl} becomes less important because strong electrostatic attractions suppress configurational entropy.

5. Supplementary References

1 Hanwell, M. D. *et al.* Avogadro: an advanced semantic chemical editor, visualization, and analysis platform. *J. Cheminf* **4**, 17/1-17 (2012).

2 Schweizer, K. & Curro, J. in *Atomistic Modeling of Physical Properties* pp319-377 (Springer, 1994).

3 Kashiwagi, Y. *et al.* Repulsive/attractive interaction among compact DNA molecules as judged through laser trapping: difference between linear- and branched-chain polyamines. *Colloid. Polym. Sci.* **297**, 397-407 (2018).

4 Takemoto, H., Ohyama, T. & Tohsaki, A. Direct sum of Coulomb potential without ambiguities of conditionally convergent series. *Prog. Theor. Phys.* **109**, 563-573 (2003).

5 Anderson, C. F. & Record, M. T. Ion distributions around DNA and other cylindrical polyions: theoretical descriptions and physical implications. *Annu. Rev. Biophys. Biophys. Chem.* **19**, 423-463 (1990).

6 Allen, M. P. & Tildesley, D. J. *Computer Simulation of Liquids*. (Oxford university press, 2017).

7 Yoo, J. & Aksimentiev, A. Improved Parameterization of Amine-Carboxylate and Amine-Phosphate Interactions for Molecular Dynamics Simulations Using the CHARMM and AMBER Force Fields. *J. Chem. Theory. Comput.* **12**, 430-443 (2016).
